# Encephalopathies with intracranial calcification in children: clinical and genetic characterization

**DOI:** 10.1186/s13023-018-0854-y

**Published:** 2018-08-16

**Authors:** Davide Tonduti, Celeste Panteghini, Anna Pichiecchio, Alice Decio, Miryam Carecchio, Chiara Reale, Isabella Moroni, Nardo Nardocci, Jaume Campistol, Angela Garcia-Cazorla, Belen Perez Duenas, Cerebral Calcification International Study Group, Giovanna Zorzi, Anna Ardissone, Tiziana Granata, Elena Freri, Federica Zibordi, Francesca Ragona, Stefano D’Arrigo, Veronica Saletti, Silvia Esposito, Chiara Pantaleoni, Daria Riva, Valentina De Giorgis, Cristina Cereda, Maria Luisa Valente, Daisy Sproviero, Maria Pilar Poo Arguelles, Carmen Fons Estupina, Anna Maria Sans Fito, Loreto Martorell Sampol, Maria Del Mar O’Callaghan Gordo, Carlos Ignacio Ortez Gonzalez, Veronica Gonzalez Alvarez, Nuria Garcia-Segarra, Carlo Fusco, Enrico Bertini, Daria Diodato, Elisa Fazzi, Jessica Galli, Luisa Chiapparini, Barbara Garavaglia, Simona Orcesi

**Affiliations:** 1 Child Neurology Unit, IRCCS Foundation C. Besta Neurological Institute, Milan, Italy; 2 Child Neurology Unit, V. Buzzi Children’s Hospital, Milan, Italy; 3 Molecular Neurogenetics Unit, Movement Disorders Diagnostic Section, IRCCS Foundation C. Besta Neurological Institute, Milan, Italy; 4 Department of Neuroradiology, IRCCS Mondino Foundation, Pavia, Italy; 5 Child Neurology and Psychiatry Unit, IRCCS Mondino Foundation, Pavia, Italy; 6 Neuropsychiatry and Neurorehabilitation Unit, IRCCS Medea, Bosisio Parini Lecco, Italy; 7 Department of Medicine and Surgery, PhD Programme in Molecular and Translational Medicine, University of Milan Bicocca, Monza, Italy; 8 Department of Child Neurology, Pediatric Research Institute, Hospital Sant Joan de Déu, University of Barcelona, Barcelona, Spain; 9 Department of Neuroradiology, IRCCS Foundation C. Besta Neurological Institute, Milan, Italy

**Keywords:** Cerebral calcification, Leukodystrophy, Aicardi-Goutières syndrome, Next generation sequencing

## Abstract

**Background:**

We present a group of patients affected by a paediatric onset genetic encephalopathy with cerebral calcification of unknown aetiology studied with Next Generation Sequencing (NGS) genetic analyses.

**Methods:**

We collected all clinical and radiological data. DNA samples were tested by means of a customized gene panel including fifty-nine genes associated with known genetic diseases with cerebral calcification.

**Results:**

We collected a series of fifty patients. All patients displayed complex and heterogeneous phenotypes mostly including developmental delay and pyramidal signs and less frequently movement disorder and epilepsy. Signs of cerebellar and peripheral nervous system involvement were occasionally present. The most frequent MRI abnormality, beside calcification, was the presence of white matter alterations; calcification was localized in basal ganglia and cerebral white matter in the majority of cases. Sixteen out of fifty patients tested positive for mutations in one of the fifty-nine genes analyzed. In fourteen cases the analyses led to a definite genetic diagnosis while results were controversial in the remaining two.

**Conclusions:**

Genetic encephalopathies with cerebral calcification are usually associated to complex phenotypes. In our series, a molecular diagnosis was achieved in 32% of cases, suggesting that the molecular bases of a large number of disorders are still to be elucidated. Our results confirm that cerebral calcification is a good criterion to collect homogeneous groups of patients to be studied by exome or whole genome sequencing; only a very close collaboration between clinicians, neuroradiologists and geneticists can provide better results from these new generation molecular techniques.

## Background

Encephalopathies with intracranial calcification constitute a group of very disabling neurological disorders. The clinical presentation is highly heterogeneous, ranging from congenital static conditions such as Adams Oliver Syndrome (AOS) [1], biphasic disorders such as Aicardi-Goutières Syndrome (AGS) [2], to severe progressive diseases like Krabbe Disease (KD) [3]. All these conditions have in common the presence of cerebral calcification on Computed Tomography (CT). Many hypotheses to explain calcium deposition in the brain have been formulated, including inflammation [4], microangiopathy [5], dystrophic process [6], abnormal metabolism of calcium [7].The identification of the molecular bases of different disorders allowed to partially clarify the pathophysiological mechanism leading to cerebral calcification in some conditions such as in disorders related to mutations in genes encoding endothelial tight junction proteins (e.g. *OCLN* and *JAM3*) [8, 9], vascular basement membrane components (*COL4A1* and *COL4A2*) [10–12], proteins involved in phosphate transport and calcium homeostasis (*SLC20A2*, one of the genes associated to Primary Familial Brain Calcification (PFBC) formerly known as Fahr’s Disease) [13]. In this study, we focused on a group of patients affected by a paediatric onset genetic encephalopathy with cerebral calcification of unknown aetiology.

## Methods

### Patients selection

We enrolled patients referred for diagnostic work-up from 2007 to 2016 to Besta Neurological Institute (Milan, Italy), Mondino Neurological Institute (Pavia, Italy) and Sant Joan de Deu Hospital (Barcelona, Spain), who presented with a childhood onset encephalopathy with cerebral calcification.

Inclusion criteria were as follows: 1) Onset before 18 years of age; 2) Cerebral Calcification documented on CT brain scan; 3) At least one performed brain MRI scan; 4) Unknown molecular diagnosis. Acquired conditions causing cerebral calcification (i.e. congenital infections, tumors) were ruled out in all patients.

### Clinical and radiological data collection

Clinical data revision focused particularly on age and signs/symptoms at onset and clinical picture at the age at genetic analysis.

Neuroradiological images were reviewed collegially by the authors (LC; AP; DT). Evaluation of MRIs included the presence and localization of cerebral calcification as well as additional cerebral alterations. Based on the type of MRI signal, we categorized white matter involvement into “demyelination”, “hypomyelination” and “delayed myelination” according to the international accepted classification [14]. We used the term “uncertain” when available data were not sufficient to discriminate between hypomyelination and delayed myelination. Cerebral calcification localization was categorized as follows: cerebral cortex, white matter, basal ganglia (including all deep supratentorial grey nuclei), cerebellum, brainstem, medulla oblongata.

### Genetic analysis

Patients were analyzed by Next Generation Sequencing (NGS) technique using a customized gene panel (Nextera Rapid Capture Custom Enrichment) containing known genes most frequently associated with diseases with cerebral calcification with childhood and adult onset (Table 1). The panel was designed with Illumina Design Studio tool. The region of interest was the gene CDS (Coding sequence) with +/− 20 bp intronic flanking region for including splicing mutations. This panel was used for library preparation; then samples were analyzed by a Miseq system (Illumina), with 50X effective mean depth. The generated reads were aligned to human genome assembly hg19 and the identified variants were annotated (Variant-Studio2.2, Illumina) and filtered, focusing on rare variants (minimum allele frequency < 1% in 1000 Genome Project [www.1000genomes.org] and ExAc [http://exac.broadinstitute.org] and Exome Sequencing Project, [http://evs.gs.washington.edu/EVS/], databases) and causing changes potentially damaging for the protein function (Polyphen2, SIFT and Mutation Taster). Conservation of alterated aminoacid was investigated with BLAST [https://blast.ncbi.nlm.nih.gov/Blast.cgi]. Sanger sequencing was performed to confirm the mutation in each patient and the segregation within the family, where possible.

**Table 1:** Genes included in our customized gene panel (Nextera Rapid Capture Custom Enrichment)

**Primary Familial Basal Ganglia Calcification (PFBC)**	**Nasu Hakola Disease**
*SLC20A2*	*TYROBP*
*PDGFB*	*TREM2*
*PDGFRB*	**Coats Plus Disease**
*XPR1*	*CTC1*
**Aicardi-Goutieres Syndrome + ** ***RNASET2***	**Inborn error of folates metabolism**
*TREX1 (AGS1)*	*FOLR1*
*RNASEH2B (AGS2)*	*SLC46A1*
*RNASEH2C (AGS3)*	*MTHFR*
*RNASEH2A (AGS4)*	*DHFR*
*SAMHD1 (AGS5)*	*MTFD1*
*ADAR1 (AGS6)*	**Occludin**
*IFIH1 (AGS7)*	*OCLN*
*RNASET2*	**Collagen IV related disorders**
**Cockayne syndrome and other DNA repair disorders**	*COL4A1*
	*COL4A2*
*DDB2*	**Hemorrhagic destruction of the brain subependymal calcification, and cataracts**
*ERCC1*	*JAM3*
*ERCC2*	**Spondyloenchondrodysplasia**
*ERCC3*	*ACP5*
*ERCC4*	**Carbonic Anhydrase Deficiency**
*ERCC5*	*CA2*
*ERCC6*	**Congenital Dyskeratosis**
*ERCC8*	*DKC1*
*GTF2H5*	*TERC*
*MPLKIP*	*TERT*
*POLH*	*TINF2*
*XPA*	*NHP2*
*XPC*	*NOP10*
**Adams Oliver syndrome**	*WRAP53*
*DOCK6*	*RTEL1*
**Friede Syndrome**	**Other calcification genes**
*AP1S2*	*CoAsy*
**Keutel Syndrome**	*PANK2*
*MGP*	*QDPR*
**Nakajo Nishimura** **syndrome**	*CYP2U1*
*PSMB8*	*GALC*
**Raine Syndrome**	*C1qB*
*FAM20C*	*ISG15*

Group of genes are captured in bold

## Results

**Fig. 1:**
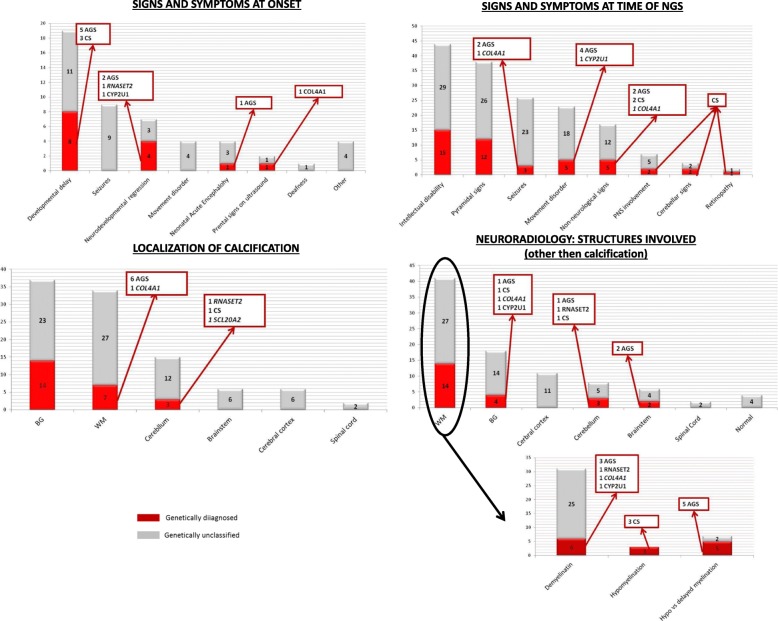
Graphical representation of clinical and radiological features of our series of patients. BG = Basal ganglia; WM = white matter

Fifty subjects (twenty-one females and twenty-nine males) were included in the study. Clinical and radiological data are summarized in Fig. 1. Genetic results are presented in Table 2. The most relevant results are described hereafter.

**Table 2:**
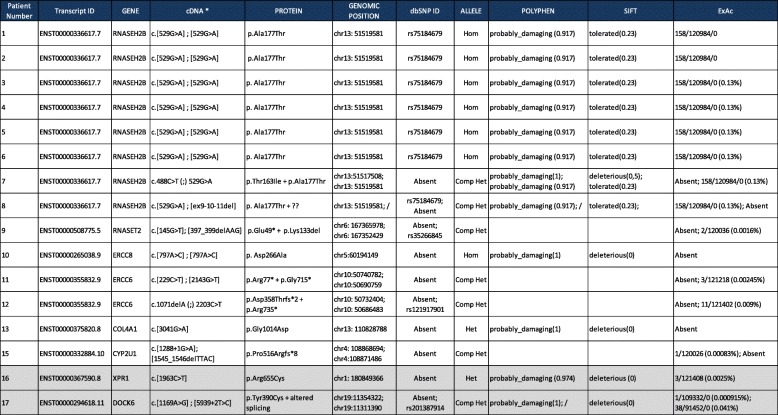
Genetic results: Found variants, in silico prediction and frequency in general population Controversial results are highlighted in grey

*Hom* Homozygous, *Het* heterozygous, *Comp* Compound

### Clinical data

The mean age of onset was 17 months (range: prenatal to 13 years; 25° percentile neonatal, 75° percentile 11 years). Patients presented the following signs or symptoms at onset developmental delay (nineteen subjects; 38%), seizures (nine; 18%), neurodevelopmental regression (seven; 14%), movement disorder (four; 8%), neonatal acute encephalopathy (four; 8%), deafness (one; 2%) other (four; 8%); the latter included psychiatric disorders, visual impairment, language delay. In two patients (4%) signs of cerebral abnormalities were already present before birth on fetal ultrasound.

The mean time between clinical onset and molecular analysis (NGS) was 10 years and 7 months (range: 10 months to 33 years; 25° percentile 5 years, 75° percentile 15 years). At the time of analysis, the clinical picture was characterized by the presence of cognitive impairment in forty-four out of fifty subjects (88%), pyramidal signs in thirty-eight (76%), seizures in twenty-six (52%), movement disorder in twenty-three (46%), non-neurological signs and symptoms in seventeen (34%), peripheral nerve involvement in seven (14%), cerebellar signs in four (8%) and retinopathy in two (4%).

### Neuroradiology

First MRI was performed at a mean age of 6 years (range: neonatal to 30 years; 25° percentile 7 months, 75° percentile 7 years). Follow-up MRI was available in thirty out of fifty patients, the meantime from the first MRI was 4 years (range 2 months to 17 years, 25°percentile 14 months 50° percentile 5 years). Beside the presence of calcification, additional MRI abnormalities were observed in forty-six out of fifty subjects. Forty-one showed white matter involvement, of which thirty-one (76%) presented a pattern suggestive of demyelination, three (7%) of hypomyelination, whereas in seven (17%) it was not possible to distinguish between hypomyelination and delayed myelination because of the too young age and absence of follow-up MRI. Basal ganglia alterations other than calcification were present in eighteen out of fifty patients (36%). These abnormalities consisted in volume loss without signal abnormalities and in the constext of a global cerebral atrophy in ten of them (55%), abnormal signal with or without atrophy in five (27%) and sign of cavitation consistent with bilalteral striatal necrosis in three (16%). Cerebral cortex was involved in eleven (22%), of which four (36%) presented cortical atrophy, four (36%) cortical malformation (polymicrogiria in all cases) and three (28%) signs of ischemic damage (watershed stroke in all the three); it is of note that in the last three patients the acquired lesions where found in the context of a more complex and likely genetic encephalopathy and therefore included in the study. Cerebellum was altered in eight out of fifty patients (16%) and brainstem in six (12%). Spinal cord MRI was performed in twenty out of fifty patients and signal abnormalities were disclosed in two.

First CT scan was performed at a mean age of 6 years (range: neonatal to 30 years; 25° percentile 8 months, 75° percentile 8 years). Follow-up CT was available in eighteen out of fifty patients, the mean time from the first CT was 4 years (range: 7 months to 13 years; 25°percentile 20 months 50° percentile 6 years). Cerebral calcification were evident in basal ganglia in thirty-seven (74%) patients, in white matter in thirty-four (68%), in the cerebellum in fifteen (30%), in the brainstem in six (12%), in cerebral cortex in six (12%), in spinal cord in two (4%).

### Clinical and genetic diagnosis

Based on clinical, radiological and laboratory findings an etiological hypothesis was suspected in thirty patients while the other twenty were classified as affected by an undetermined condition. NGS analysis provided positive results in sixteen out of fifty patients (32%) leading to a definite etiological diagnosis in fourteen out of sixteen. Variants of uncertain significance were found in two subjects.

Thirteen out of thirty with suspected diagnosis and only one out of twenty classified as undetermined condition finally received a genetic diagnosis. The genetic diagnosis was consistent with the clinical hypothesis in all but one who had been clinically diagnosed as AGS and then found to carry biallelic mutations in *RNASET2* (more details are given below)*.*

The most frequent diagnosis in our series was AGS which is the most frequent leukodystrophy with cerebral calcification. Diagnosis of AGS repose on a number of clinical, radiological and biological criteria [15] (Table 3). Sixteen patients were suspected to suffer from AGS, nine received genetic confirmation. Twelve out of the sixteen suspected AGS fulfilled classic diagnostic criteria, in eight of them mutations in the seven known AGS-genes were found, while one was the *RNASET2* mutated patient and three were finally diagnosed as AGS mutation negative. Four patients fulfilled only partially diagnostic criteria of AGS, one of them carried biallelic *RNASEH2B* mutations and received a diagnosis of atypical AGS, the other three remained unclassified.

**Table 3:** AGS diagnostic criteria

1	Early onset encephalopathy with psychomotor delay, spasticity, extrapyramidal signs and microcephaly, the latter appearing in the course of the first year of life.
2	Calcifications particularly visible at basal ganglia level (putamen, pallidus and thalamus), but also extending to the periventricular white matter.
3	Cerebral white matter abnormalities.
4	Cerebral atrophy.
5	Exclusion of pre−/perinatal infections, in particular the TORCH complex (toxoplasmosis, rubella, cytomegalovirus, herpes simplex virus).
6	Chronic lymphocytosis (> 5 cells/mm3) on CSF examination, not accompanied by any other sign of an infectious process.
7	Raised INF-alpha in the CSF (> 2 IU/ml).
8	Elevated neopterins and biopterins in CSF, sometimes associated with decreased folates.
9	Important systemic symptoms in the early stages of the disease include irritability, feeding and sleeping difficulties, unexplained fevers and the appearance of chilblain-like skin lesions on the fingers, toes and ears.
10	Genetic screening for mutations in the seven genes known to cause AGS allows definitive confirmation of the diagnosis in the majority (95%) of cases.

Criteria 1–5 plus criteria 6 or 7 were considered necessary to estabilish the clinical diagnosis of AGS. Criteria 8–10 were considered supportive criteria

In two patients PFBC was suspected both remained unclassified.

**Fig. 2:**
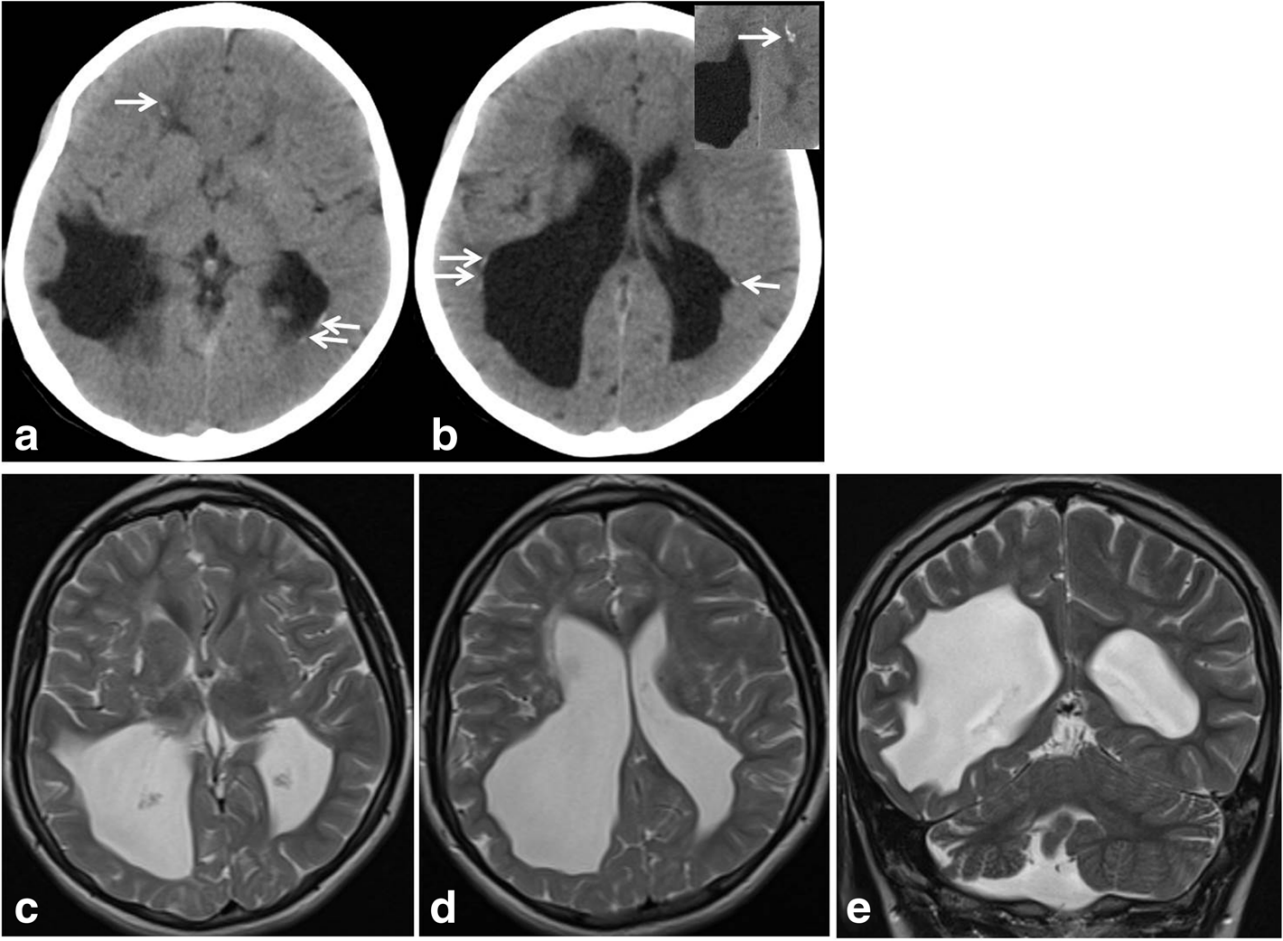
*COL4A1*-related leukoencephalopathy –Brain CT, **a** and **b** and brain MRI, **c**-**e**, show «ex vacuo» enlarged lateral ventricles with irregular profiles mainly posteriorly and on the right associated with lacunar infarctions in the right basal ganglia. CT demonstrate small sub-ependymal calcification and insert in **b** show calcification in the sub-cortical white matter (arrows). Coronal image in E demonstrate right posterior cranial fossa and right cerebellar hemisphere hypoplasia

**Fig. 3:**
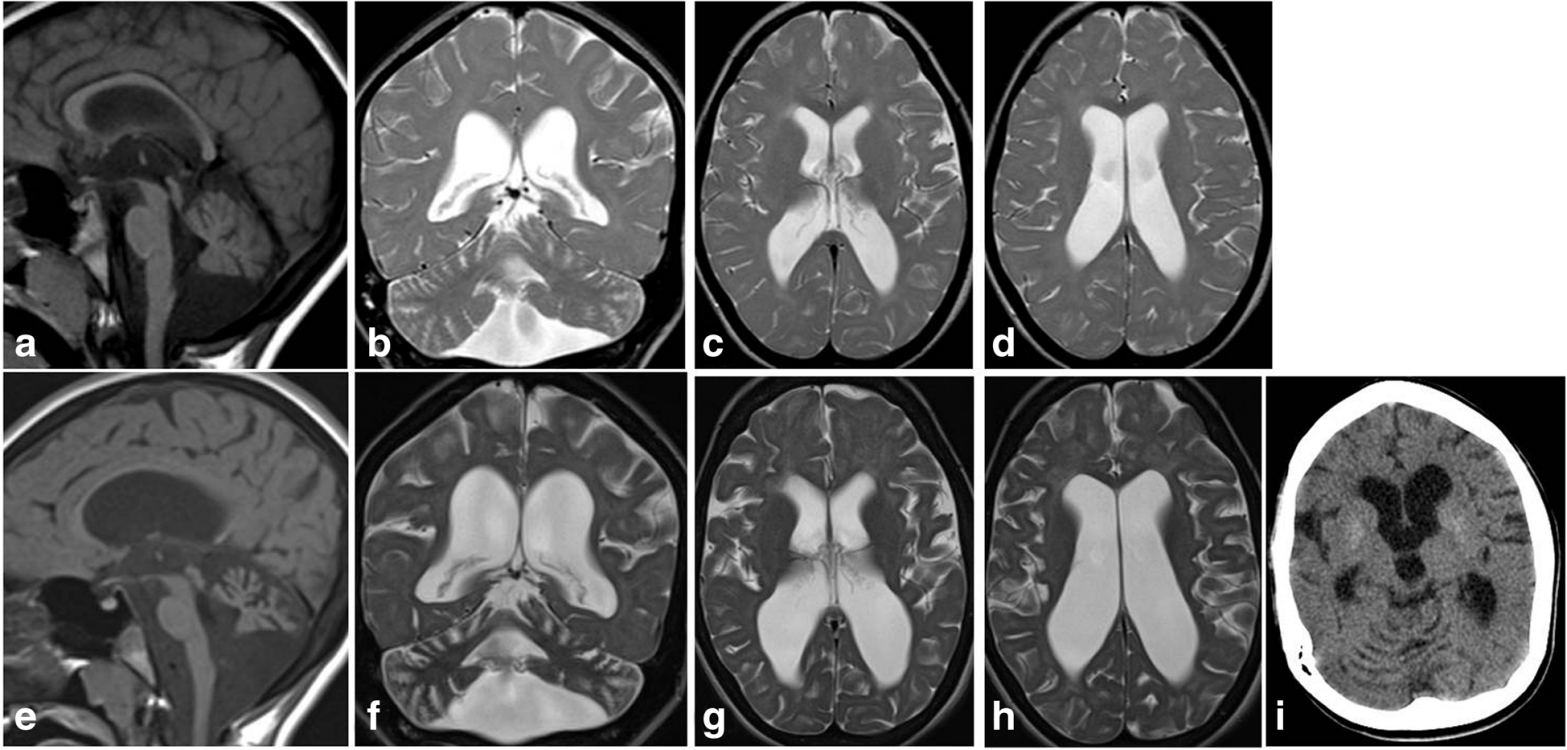
Cockayne syndrome –Brain MRI at age 5 years, top (**a**-**d**), brain MRI at age 9 years, bottom (**e**-**h**), and brain CT at age 9, (**i**). Note the diffuse cerebral atrophy progression, mainly in the posterior fossa, and the diffuse slight white matter hyperintensity due to hypomyelinating leukoencephalopathy. CT demonstrate faint hyperdensity in the putamina due to fine calcification

Two patients presented a clinical and radiological picture suggesting a Collagen IV- related disorder (Fig. 2), one has been confirmed while in the other no pathogenic mutations have been found. Three patients was suspected to suffer from Cockayne Syndrome (Fig. 3), NGS confirmed the clinical hypothesis in all.

One patient was suspected to suffer from Leukodystrophy with Cysts and Calcification (LCC), two from *ADAR1-*related striatal degeneration, one from Occludin-related condition, one from Adams Oliver Syndrome, one from Revesz Syndrome. In none of these 6 patients pathogenic mutations have been found. It is of note that when our gene panel has been designed the molecular cause of LCC was still obscure. Morover *DOCK6* was the only AOS-gene included in the panel because of limited space availability and because it was the one most commonly associated with cerebral calcification.

Among the twenty-eight patients in which no etiological hypothesis was formulated, pathogenic mutations were found in only one. She was the young woman suffering from *CYP2U1-*related condition.

#### Atypical AGS (patient 6, Fig. 4)

This 13 year-old boy presented at 9 months of age with subacute onset of neurodevelopmental regression signs of pyramidal and extrapyramidal involvement, and evolved to a spastic-dystonic tetraplegia with global hypokinesia but with a good social interaction. He never developed microcephaly or extraneurological signs. Serial MRIs showed the presence of non -progressive multifocal white matter abnormalities and multiple CT scans revealed non progressive bilateral spots of calcification in basal ganglia. Cerebrospinal fluid (CSF) cell count tested at 14 months, CSF Interferon alpha tested at 3 years, Interferon stimulated Genes analysis (interferon signature) tested at 12 years, were all normal. Despite not all criteria for the diagnosis of AGS were fulfilled NGS disclosed a common missense mutation of *RNASEH2B* in a homozygous state. Given the presence of some un common findings (uncommon MRI pattern [16] and normal CSF cells counts close to clinical onset) a diagnosis of atypical AGS was done.

**Fig. 4:**
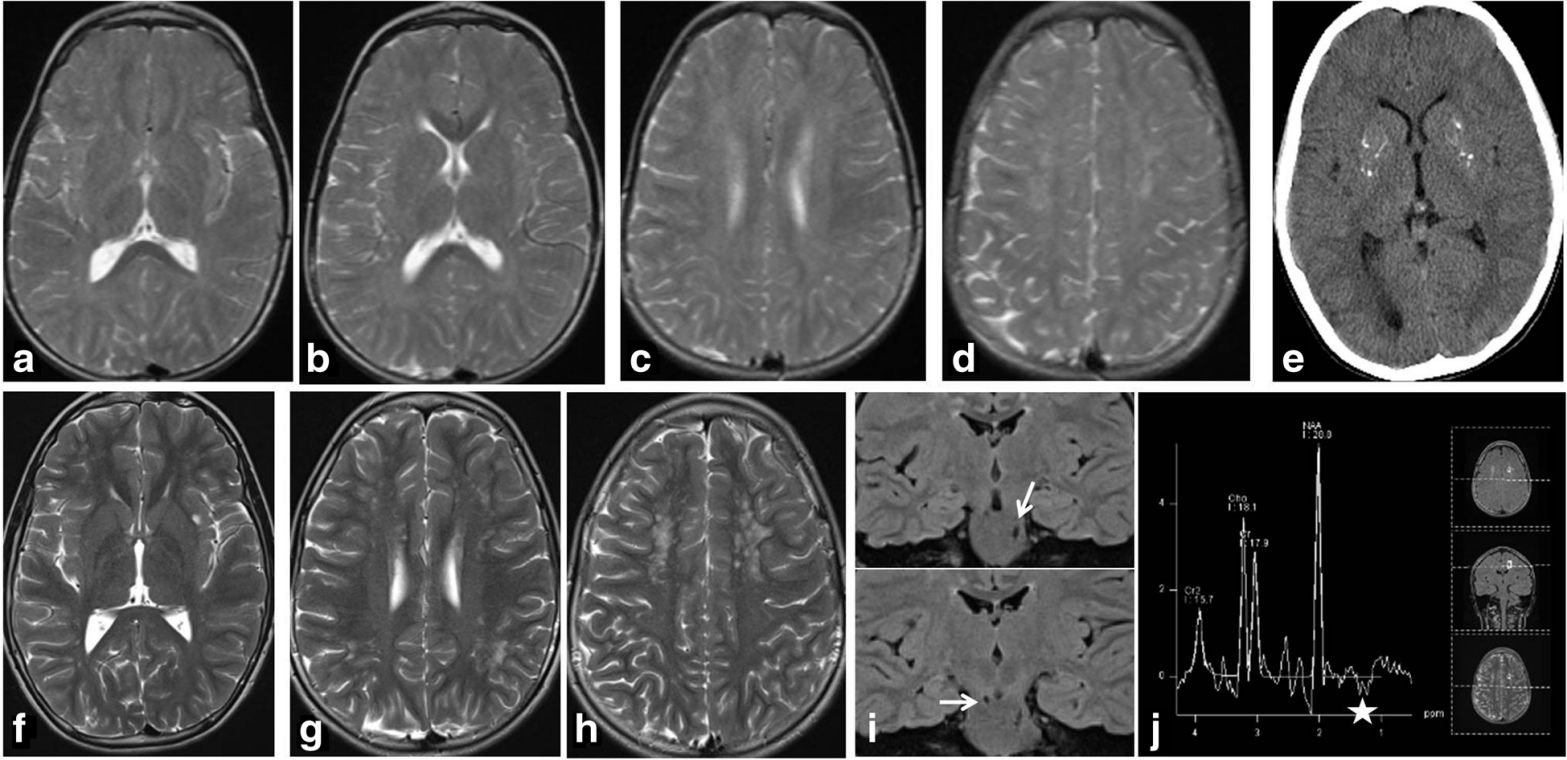
Atypical AGS – Top, brain MRI at 4 years old, (**a**-**d**), demonstrate mildly hyperintense T2 signal of the white matter relative to the cortex probably due to delayed myelination; CT show (**e**) basal ganglia calcification. Bottom, (**f**-**j**), brain MRI at 11 years old demonstrate myelination progression and multiple small confluent T2 hyperintensities in the sub-cortical areas mainly in F1. Enlarged perivascular spaces are visible in the brainstem (arrows in **i**); MRS in **j** shows lactate (asterisk) in the abnormal frontal white matter

#### RNASET2-related leukodystrophy

Patient 9 was diagnosed as affected by *RNASET2-*related leukodystrophy. The clinical and radiological picture was quite different compared to the one described in this condition and overlapped significantly with AGS as we described elsewhere [17].

#### CYP2U1-related encephalopathy (Fig. 5)

**Fig. 5:**
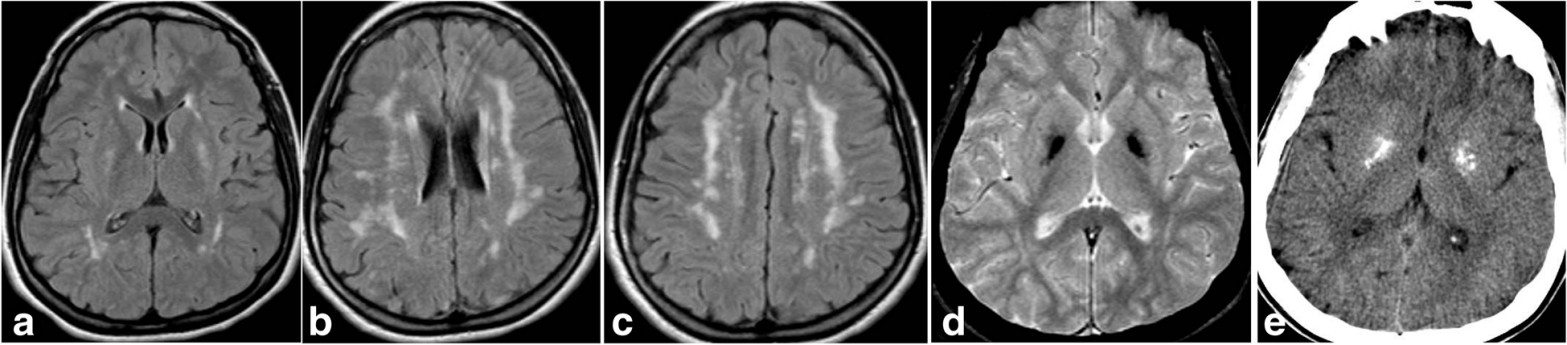
CYP2U1-related disorder- Brain MRI at 25 years old, FLAIR images in (**a**-**c**) and FFE image in (**d**), show bilateral hyperintensities in the putamina and confluent marked hyperintensities in the sub-cortical regions of cerebral hemispheres. In **d** and in **e** (CT), bilateral pallidal marked hypointensity and hyperdensity due to calcification is seen. Examination performed 7 years later was unchanged (data not shown)

In patient 15 biallelic mutations of *CYP2U1* were found. This case has previously been reported by Iodice et al. [18] and was characterized by onset at the end of the first year of life of a slowly progressive encephalopathy with spastic tetraplegia, mild upper limb dystonia and borderline cognitive performance. An interesting non-reported finding was the presence on MRI not only of spotty multifocal white matter abnormalities and pallidal calcification but also of bilateral putaminal T2 hyperintensisty, (Fig. 5) which has never been reported in *CYP2U1*–related condition so far. Given the complex clinical and radiological picture of this and other reported patients [19] we propose to consider *CYP2U1* mutations as the cause of a broader phenotypic spectrum ranging from pure spastic paraparesis (SPG56) to a veritable encephalopathy with cerebral calcification.

### Uncertain genetic results

In 2 patients NGS analysis demonstrated ambiguous results.

In patient 17 biallelic mutations were found in *DOCK6,* one of the genes of Adams Oliver Syndrome (AOS) [1]. Our patient did not show the clinical presentation of AOS, but conversely she fulfilled clinical criteria for Aicardi-Goutières Syndrome.

The second patient (patient 16) was found to carry a mutation in a PFBC-related gene: *XPR1*. This is a 9 year-old boy that presented at 3 years of age with acute onset of generalized dystonia shortly after an intercurrent viral infection. MRI showed bilateral striatal lesions hyperintense on T2wi associated with bilateral striatal calcification. At present we can just report these observations, more data are needed and functional studies are ongoing in order to explore the actual role of the mutations.

### Genetically unclassified patients

**Fig. 6:**
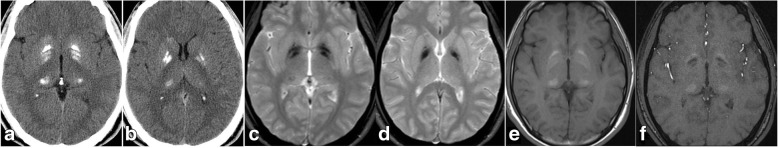
PFBC – Brain CT (**a**, **b**) and MRI (**c**-**f**) at 17 years old show bilateral coarse calcification in globus pallidus and thalamus. Note the different signal of calcification on T2 FFE, **c** and **d**, T1-weighetd, **e**, and time-of-flight (TOF, **f**) images

In thirtyfour patients genetic analysis resulted negative. These included seven patients who fulfilled criteria for a specific clinical diagnosis [three AGS, one AOS, one PFBC (Fig. 6), one Revesz Syndrome, one LCC] confirming the genetic heterogeneity underlying some peculiar clinical syndromes and the partial knowledge of their genetic bases, even in the “genome” era.

In the group of negative patients is included patient 14, a girl now aged 16 affected by a classical Down Syndrome. From the age of 14 years she started to present akynetic-rigid parkinsonism with tremor in the upper limbs. She underwent an MRI showing bilateral rock-shaped calcification in the pallidal nuclei, putamina and dentate nuclei. Pallidal calcification in Down Syndrome has been reported previously and interpreted as a sign of premature aging [20]. The clinical and radiological picture in our case was quite peculiar and prompted us to widen the molecular differential diagnosis leading to the identification the unreported variant in one of the PFBC-related gene, namely *SLC20A2* c.[1301C > G]. The variant was predicted to be “disease causing” in silico (Polyphen-2, SIFT, MutationTaster). Segregation analysis revealed that the healthy father carried the same variant. He underwent a cerebral CT scan that resulted normal. It is difficult to ascertain if cerebral calcification were only related to 21 trisomy or if the concomitant presence of the *SLC20A1* variant played also a role.

In the group of unclassified patients we were able to identify some subset of patients sharing similar peculiar phenotypes. This was the case of a previously reported patient affected by a leukoencephalopathy with spinal cord calcification of unknown genetic etiology [21]. A second patient with a strikingly similar phenotype was present in our series and we recently identified a third patient outside the study sample. Based on the similar phenotype, we started looking for a possible common genetic abnormality and this approach led to the identification of the genetic cause of the disease [22].

## Discussion and conclusions

We here describe the clinical and radiological features of a series of fifty patients affected by a pediatric onset encephalopathy with cerebral calcification. Age at onset was quite variable but the majority of patients presented during the first 2 years life. Intellectual disability and pyramidal signs were commonly observed, while movement disorders and epilepsy were less frequent. Cerebellar signs, peripheral nerve and retinal involvement were rare. They were present in 3 subjects and they have been essential hints to reach the etiological diagnosis of CS.

In our series, white matter involvement was present in the majority of patients. A demyelinating pattern was the most frequently observed. Hypomyelination was present only in patients affected by CS while among subjects with “uncertain” pattern, AGS was the only diagnosis, confirming that inadequate myelination can be observed in AGS patients [16]. Only one subject presented a clinical picture consistent with isolated dystonia but associated to bilateral pallidal calcification. Dedicated gene panels for primary and secondary movement disorders did not allow a definite diagnosis in this subject.

The most frequent localization of cerebral calcification was basal ganglia. However, movement disorders were observed only in a minority of patients suggesting that, at least in some cases, calcifications could be just an epiphenomenon of the disease without a significant role in the genesis of clinical signs and symptoms.

AGS was the most frequent diagnosis and *RNASEH2B* was the most commonly involved gene*.* This result is in line with literature where AGS is described as the most common pediatric onset encephalopathy with cerebral calcification [23] and *RNASH2B* as the gene most commonly involved [2].

In our study genetic analysis and deep phenotyping were strictly connected. The presence of cerebral calcification represented the criterion for selecting the initial group of patients. Subsequently a very careful clinical work of investigating relevant clinical and radiological signs was the crucial point that resulted extremely important in analyzing NGS data. It was indeed the central point in order to address the attention to specific genes and to evaluate the significance of the various variants found by the customized panel. This approach led us to solve a considerable number of cases (32%). Moreover we made a rapid diagnosis in patients affected by classic phenotypes of diseases that can be caused by different genes, such as AGS, or by large genes such as *COL4A1* and *COL4A2*. In fact in both these situations traditional Sanger sequencing appears to be obsolete, time consuming and not cost-effective. Our results suggest that all patients presenting cerebral calcification should undergo first of all to an attentive and deep phenotyping process done by a qualified team of experienced professionals. If this leads to a clinical hypothesis the next step is to perform customized gene panel except in the case of AGS where the common mutation p.Ala177Thr in *RNASEH2B*/*AGS2* should be tested before, at least in patients with Italian background. Whole exome sequencing should be chosen as first line genetic analysis in the other patients.

As we said cerebral calcification represent a useful diagnostic hint, but they should be carefully evaluated when found in advanced disease stages because in these cases it could become a confounding factor. Calcium deposition indeed can be secondary to white matter degeneration and can be observed in advanced stages of some leukodystrophies not usually characterized by cerebral calcification such as X-linked adrenoleukodystrophy [24–26] or Alexander disease [27–30]. This was the case of one patient of our series who was referred for diagnostic work-up of a severe leukodystrophy with cerebral calcification. When CT was performed and calcification was found she presented an advanced stage of her progressive condition. Gene panel for cerebral calcification resulted negative and the girl was lately diagnosed as affected by D-bifunctional protein deficiency (DBPD). As far as we know cerebral calcification has not been reported so far in patients affected by DBPD and it probably represent an epiphenomenon of severe white matter degeneration instead of an unreported feature of the disease.

Cerebral calcification was studied by CT in all patients included in our series. In clinical practice CT has almost uniformly been substituted by MRI and literature report controversial data about the best technique to be used to evaluate the presence of cerebral calcification [31–33]. From one side it seems that CT is more powerful for the identification of small calcification [33] such as those observed in *COL4A1*-related disorder, but at the same time it has to take into account the risk related to exposure to ionizing radiation [34]. More studies will be necessary to solve this problem. From our perspective, and as it appears clear from our series, in all patients presenting a genetic encephalopathy of unknown aetiology cerebral calcification should be looked for. At current state of things we deem that an attentive evaluation by MRI specific sequences should be initially chosen. If MRI is uninformative low-dose CT should be performed as second option.

With our study we identified new phenotypes associated with already known genes (i.e. *RNASET2*), but also patients presenting classical phenotypes who tested negative for all known genes and therefore good candidates for exome or whole genome sequencing.

Lastly, management of patients in a restricted number of referral centers and the strict multidisciplinary collaboration among these centers allowed identifying unrelated patients with similar clinical and radiological phenotypes. This led to identify new syndromic entities and their molecular bases. Our approach confirms that only a very close collaboration between clinicians, neuroradiologists and geneticists can provide better results from the new generation molecular techniques.

## Abbreviations

AGS: Aicardi-Goutières syndrome
AOS: Adams Oliver Syndrome
CDS: Coding sequence
CS: Cockayne syndrome
CSF: Cerebrospinal Fluid
CT: Computed Tomography
KD: Krabbe disease
NGS: Next Generation Sequencing
PFBC: Primary Familial Brain Calcification
